# Supplementary material for: SPACEL: deep learning-based characterization of spatial transcriptome architectures
Source: Nat Commun. 2023 Nov 22;14:7603. doi: 10.1038/s41467-023-43220-3 (PMC10663563; doi:10.1038/s41467-023-43220-3)
Supplement: Supplementary file 1 — Supplementary Information [file 41467_2023_43220_MOESM1_ESM.pdf]

Supplementary Figures for “SPACEL: deep-learning based characterization of spatial transcriptome architectures”

Supplementary Figure 1

a

Method	Statistical model-based	Pseudo-spot simulation	Deep learning-based	Eliminate variation between ref. and ST
Spoint	Y	Y	Y	Y
Cell2location	Y	N	N	Y
CARD	Y	N	N	Y
RCTD	Y	N	N	N
Tangram	N	N	Y	N
DestVI	N	N	Y	Y
SpatialDWLS	N	N	N	N
Stereoscope	Y	N	N	N
Seurat	N	N	N	N
DSTG	N	Y	Y	Y
STRIDE	N	N	N	N

b

Method	Type of input	Deep learning-based	Multiple slices	Batch Correction
Splane	Cell-type composition	Y	Y	Adversarial loss
STACI	Gene expression	Y	Y	Over-parameterizetion
STAligner	Gene expression	Y	Y	Triplet loss
PRECAST	Gene expression	Y	Y	Batch effect projection
STAGATE	Gene expression	Y	N	NP
SpaGCN	Gene expression	Y	N	NP
BayesSpace	Gene expression	N	N	NP
stLearn	Gene expression	N	N	NP

c

Method	Alignment strategy	Robust to shape variation	Global optimal alignment	Automatic flip slices
Scube	Global optimization	Y	Y	Y
PASTE	Optimal transport	N	Y	Y
STAligner	ICP algorithm	Y/N	Y/N	N

Supplementary Figure 1. Comparison of supported features of method with similar function as SPACEL modules. a, Comparison of supported features across Spoint and other cell type deconvolution methods. **b**, Comparison of supported features across Splane and other spatial domain identification methods. **c**, Comparison of supported features across Scube and other 3D tissue alignment methods.

Supplementary Figure 2

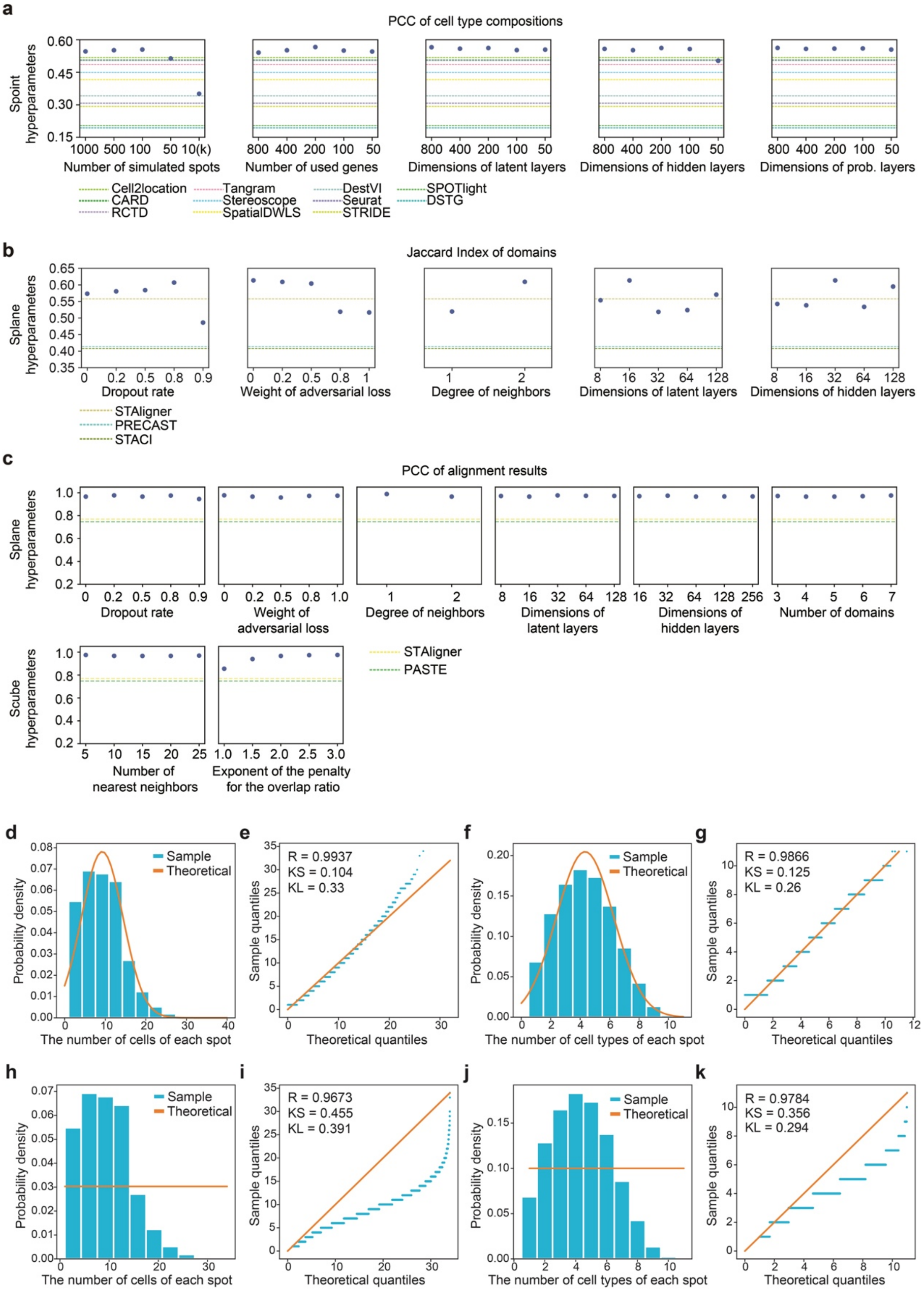

Supplementary Figure 2. Performance of SPACEL modules variations in hyperparameter settings or specific design choices. a, Average PCC values of Spoint deconvolution results under different Spoint hyperparameters for ST datasets with single cell resolution across all cell types. In each panel, the dashed lines represent results from other methods. Blue points represent the results of hyperparameter tuning. PCC, pearson's correlation coefficient. **b,** Average Jaccard indexes between the cortical layers/WM annotated by the original study and the spatial

domains identified by Splane under different Splane hyperparameters for the DLPFC dataset across all slices. WM, white matter. **c,** Average PCC values of Scube's alignment results under different Splane and Scube hyperparameters for the STARmap dataset with a crop ratio equal to 0.25 across all slices. **d,f,** Density histogram of the number of cells (d) and the number of cell types (f) in each spot and the density curve fitted with a normal distribution. The spots are formed by aggregating cells in MERFISH data of human brain tissue. **e,g,** Quantile-quantile plot of the number of cells (e) and the number of cell types (g) fitted with a normal distribution, where the orange line (y=x) represents a perfect fit. The three metrics in the plot, R (Pearson correlation coefficient), KS (Kolmogorov-Smirnov test statistic), and KL (Kullback-Leibler divergence), are used to measure the goodness of fit between the real data and the fitted values. **h,j,** Density histogram of the number of cells (h) and the number of cell types (j) in each spot and the density curve fitted with a uniform distribution. **i,k,** Quantile-quantile plot of the number of cells (i) and the number of cell types (k) fitted with a uniform distribution. Source data are provided as a Source Data file.

Supplementary Figure 3

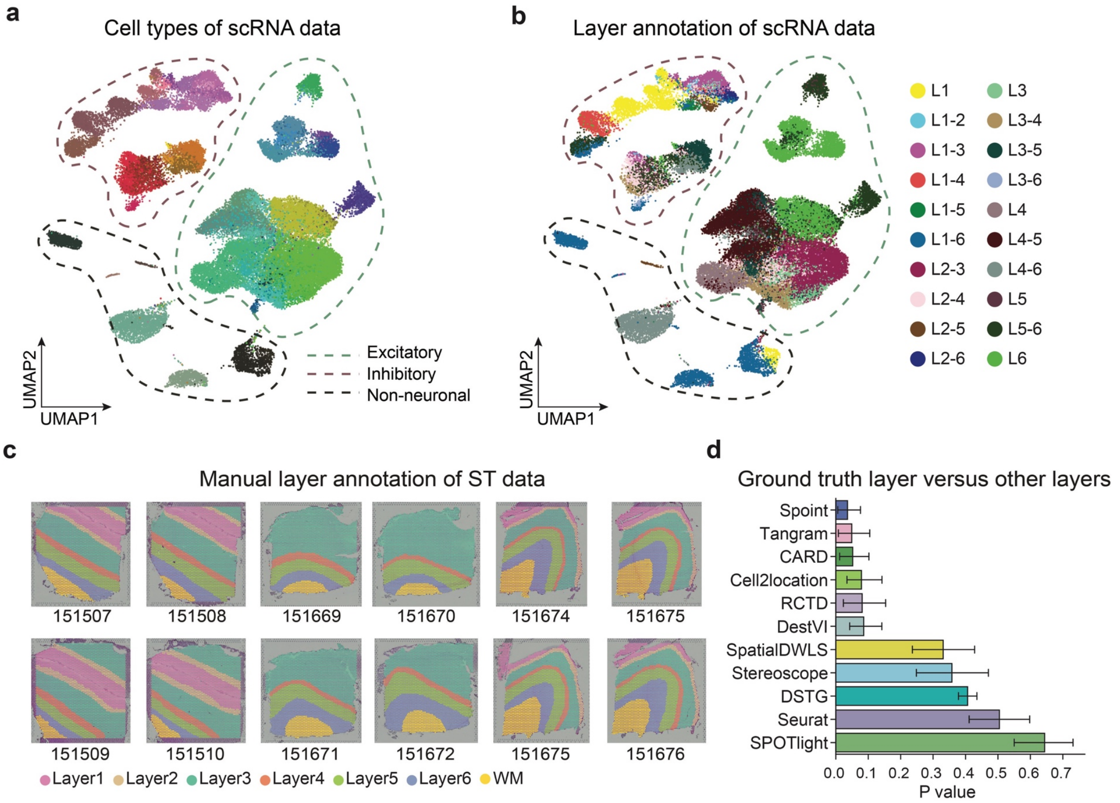

Supplementary Figure 3. Evaluation of Spoint's performance in human DFPLC Visium dataset. a,b, Uniform manifold approximation and projection (UMAP) of all cells from the human brain scRNA-seq dataset downloaded from the Allen Brain Map, colored with the cell types (a) and the cortical layers (b) annotated by the original study (Hodge *et al.* ***Nature*** 573, 7772, 2019). **c,** Cortical layer 1~6 and white matter (WM) for the 12 ST slices of the DLPFC dataset, annotated by the original study (Maynard *et al.* ***Nat. Neurosci.*** 24, 425, 2021). **d,** P-values from the two-sided Wilcoxon rank-sum test of the deconvolution results generated by Spoint and other deconvolution methods for the DLPFC dataset. Bar heights, mean values; whiskers, mean values ± 95% confidence intervals; n=56 cell types. Source data are provided as a Source Data file.

Supplementary Figure 4

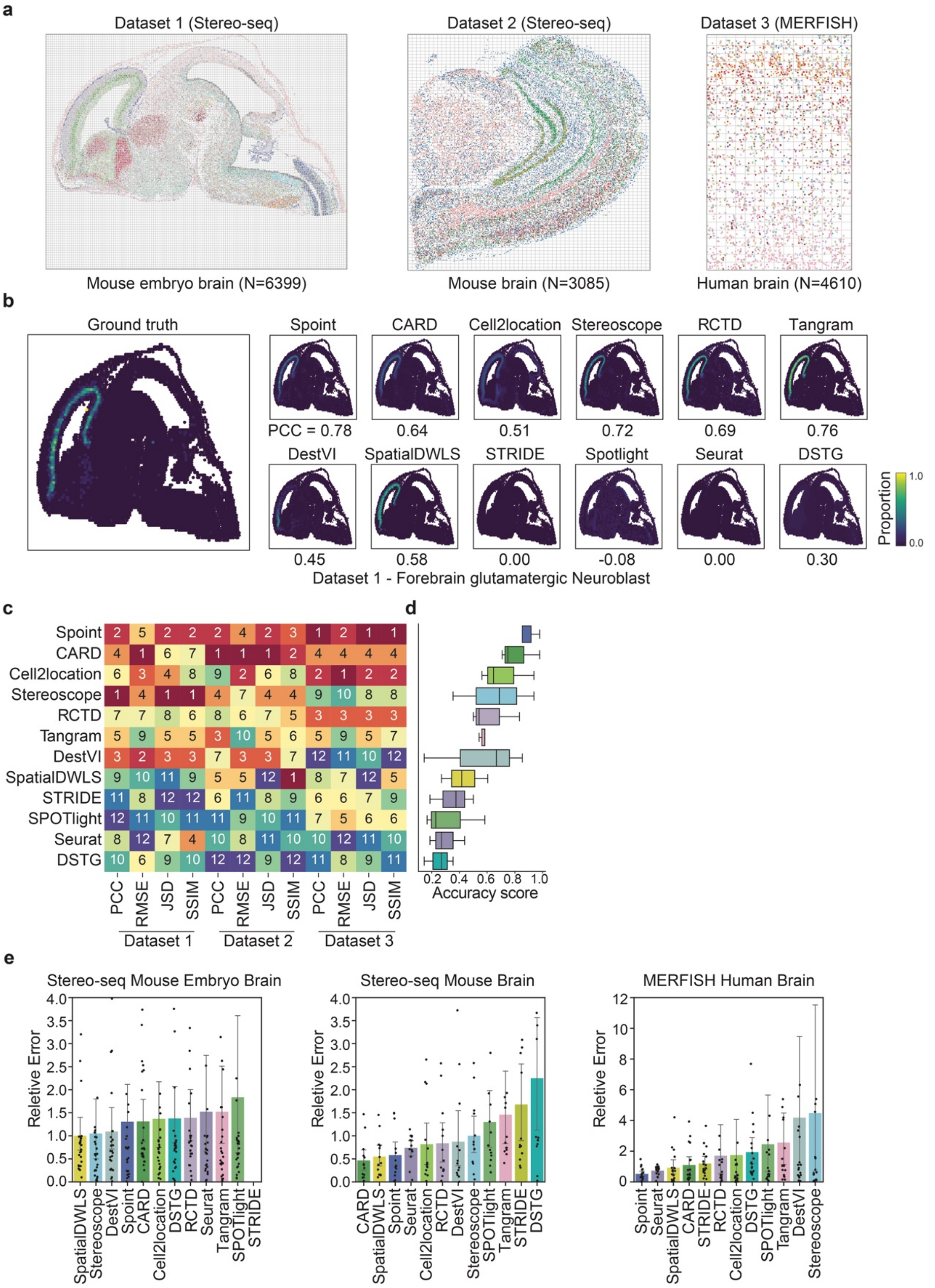

Supplementary Figure 4. Benchmarking of Spoint's performance in real ST datasets with single cell resolution. a, Three datasets (Mouse embryo brain: Stereo-seq; Mouse brain: Stereo-seq; Human brain: MERFISH) with cells annotated by cell types. Each grid represents a simulated spot containing multiple cells. **b,** The proportion of Forebrain glutamatergic Neuroblast in the spots from Dataset 1, including the ground truth and the predicted results of 12 deconvolution methods. **c,** Heatmaps illustrating the rankings of each deconvolution method based on PCC, RMSE, JSD, and SSIM metrics across three datasets. PCC, pearson's correlation coefficient; RMSE,

root mean square error; JSD, Jensen-Shannon divergence; SSIM, structural similarity index measure. **d,** Box plots of accuracy score of the 12 methods for all the three datasets. Center line, median; box limits, upper and lower quartiles; whiskers, 1.5× interquartile range; n = 12 metrics on three independent datasets. **e,** Bar plots of relative error (the ratio of predicted cell type proportion summed over all spots versus ground truth) of each deconvolution methods in Stereo-seq mouse embryo brain (n=27 cell types), Stereo-seq mouse brain (n=13 cell types), and MERFISH human brain (n=18 cell types) dataset. STRIDE is not available in Stereo-seq mouse embryo brain dataset. Bar height, mean value; whiskers, mean values ± 95% confidence intervals. Source data are provided as a Source Data file.

Supplementary Figure 5

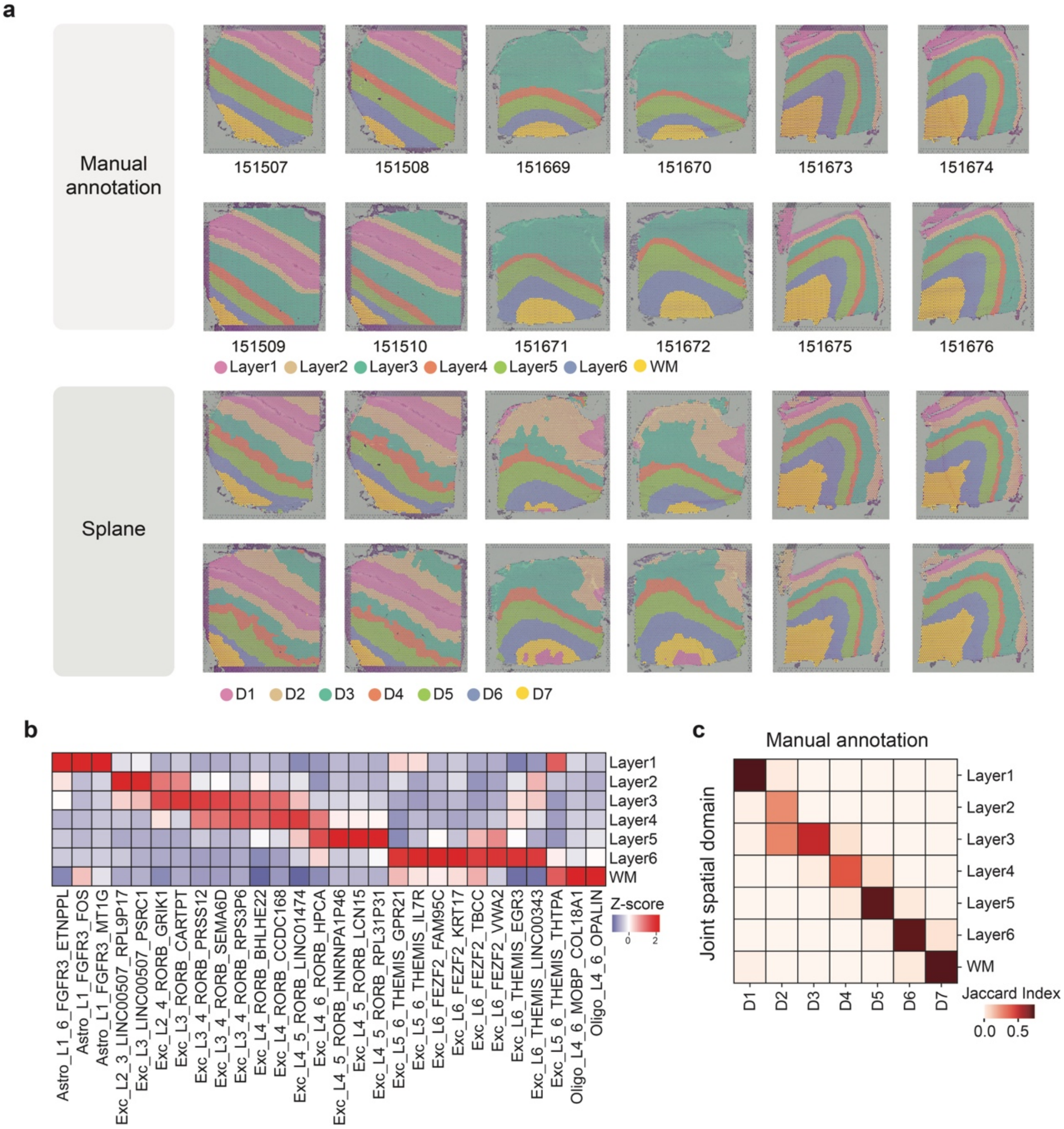

Supplementary Figure 5. Spatial domain identification by Splane in human DFPLC Visium dataset. a, Cortical layer 1~6/WM annotated by the original study (Maynard *et al.* ***Nat. Neurosci.*** 24, 425, 2021; upper panels) and spatial domains identified by Splane (lower panels) for the 12 ST slices of the DLPFC dataset. WM, white matter. **b,** Scaled proportion of each cell sub-type predicted by Spoint in cortical layers 1-6 and WM. Cell type with moran index more than 0.2 was shown. **c,** Jaccard indexes between the cortical layers/WM annotated by the original study and the spatial domains identified by Splane. Source data are provided as a Source Data file.

Supplementary Figure 6

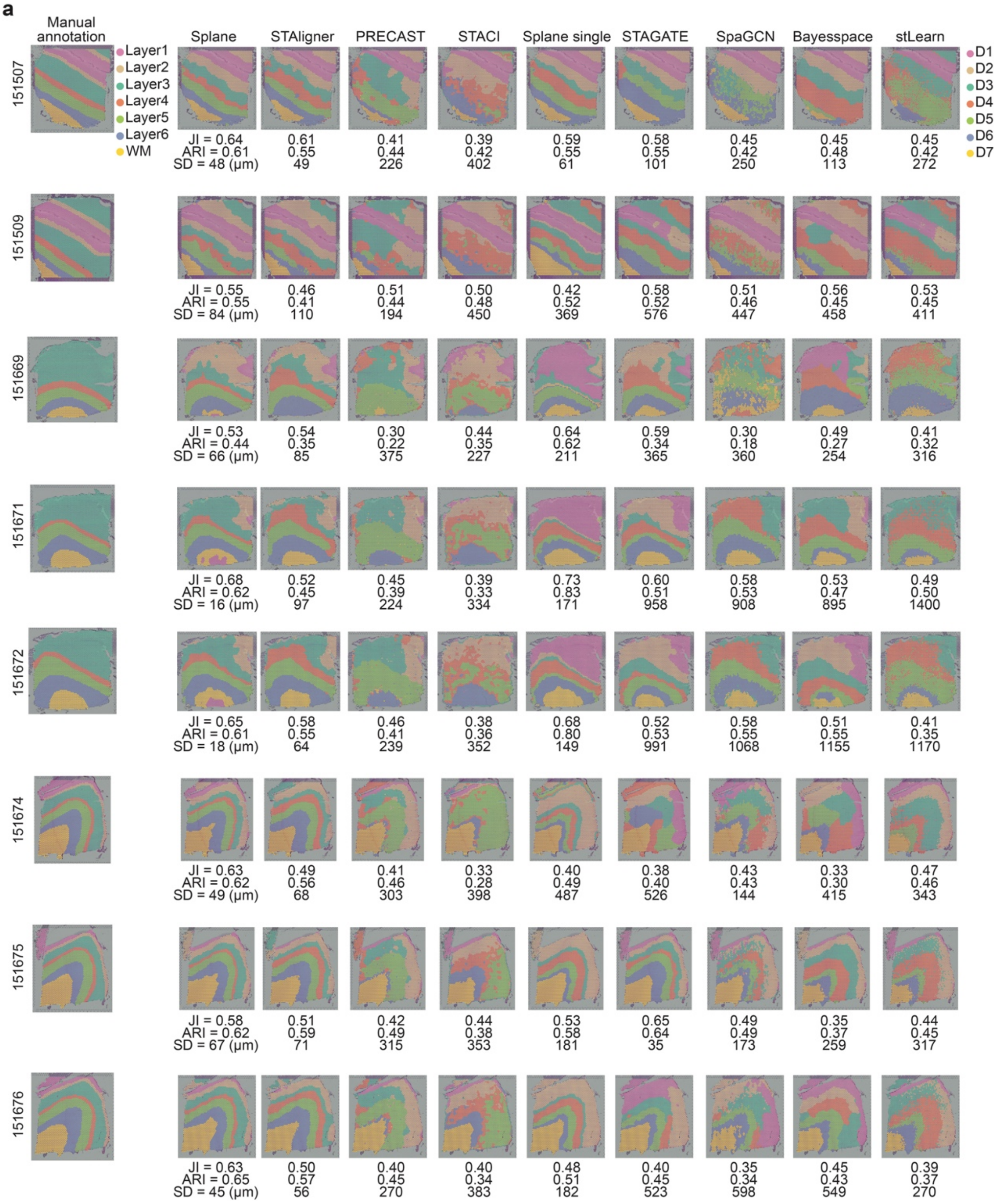

Supplementary Figure 6. Spatial domain generated by methods for multi-slice analysis in human DFPLC Visium dataset. From left to right are Cortical layer 1~6/WM annotated by the original study (Maynard *et al.* ***Nat. Neurosci.*** 24, 425, 2021), spatial domains identified by Splane, STAligner, PRECAST, STACI, Splane-single, STAGATE, SpaGCN, Bayesspace, and stLearn for the slices 151507, 151509, 151669, 151671, 151672, 151674, 151675, and 151676. JI, Jaccard index; ARI, Adjusted Rand Index; SD, shifting distance. Source data are provided as a Source Data file.

Supplementary Figure 7

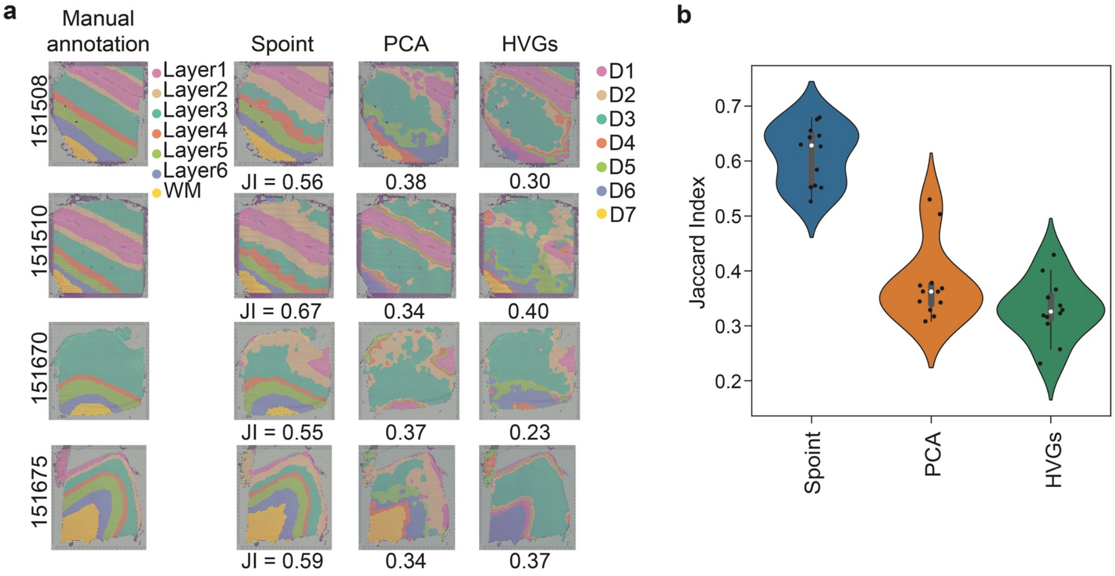

Supplementary Figure 7. Comparison of spatial domain identified by Splane using the gene expression as input. a, Comparison of spatial domains identified by Splane using the deconvolution results from Spoint, PCA matrix from normalized highly variable genes (HVGs) expression, and normalized HVGs expression matrix as input for slice 151508, 151510, 151670, and 151675. Layer1~Layer6, cortical layer 1~6; WM, white matter; JI, Jaccard index; PCA, principal component analysis. **b,** Violin plots of Jaccard indexes between cortical layers and corresponding spatial domains identified by Splane using the deconvolution results from Spoint, PCA matrix from normalized HVGs expression, and normalized HVGs expression matrix as input. Center line, median value; box limits, upper and lower quartiles; whiskers, 1.5× interquartile range; n=12 slices. Source data are provided as a Source Data file.

Supplementary Figure 8

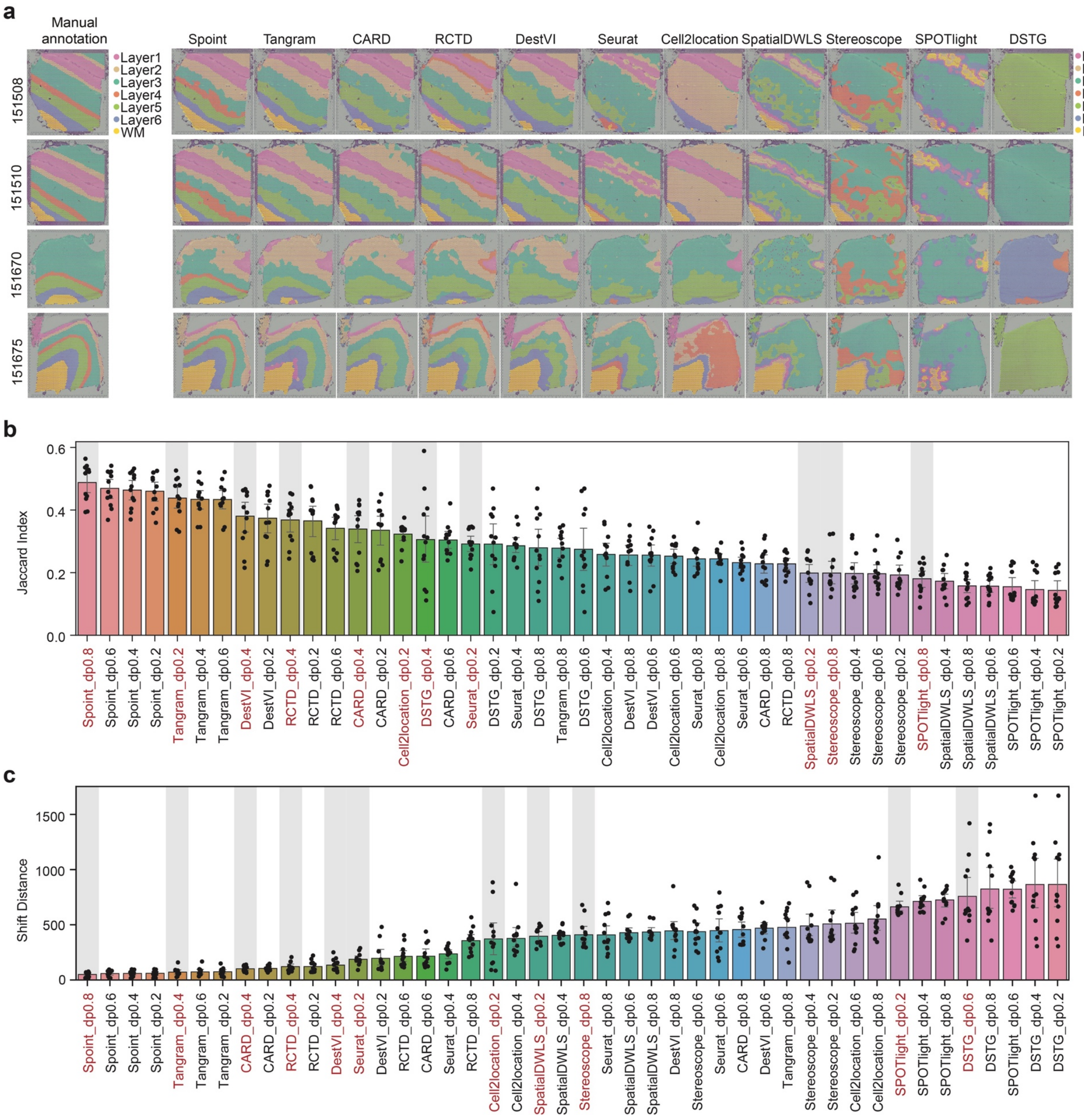

Supplementary Figure 8. Comparison of spatial domain identified by Splane using the cell type composition predicted by other deconvolution methods as input. a, Comparison of spatial domains identified by Splane using the deconvolution results from Spoint, Tangram, CARD, RCTD, DestVI, Seurat, Cell2location, SpatialDWLS, Stereoscope, SPOTlight, and DSTG as input for slice 151508, 151510, 151670, and 151675. Layer1~Layer6, cortical layer 1~6; WM, white matter; JI, Jaccard index. **b,c,** Bar plots of Jaccard indexes (b) and Shift Distances (c) of using 12 deconvolution methods as input for different hyperparameter settings of dropout rate (dp: 0.2, 0.4, 0.6, and 0.8). The gray background represents the selected hyperparameter based on the best-performing results for each deconvolution method. Bar height, mean value; box limits, upper and lower quartiles; whiskers, mean values ± 95% confidence intervals; n=12 slices. Source data are provided as a Source Data file.

Supplementary Figure 9

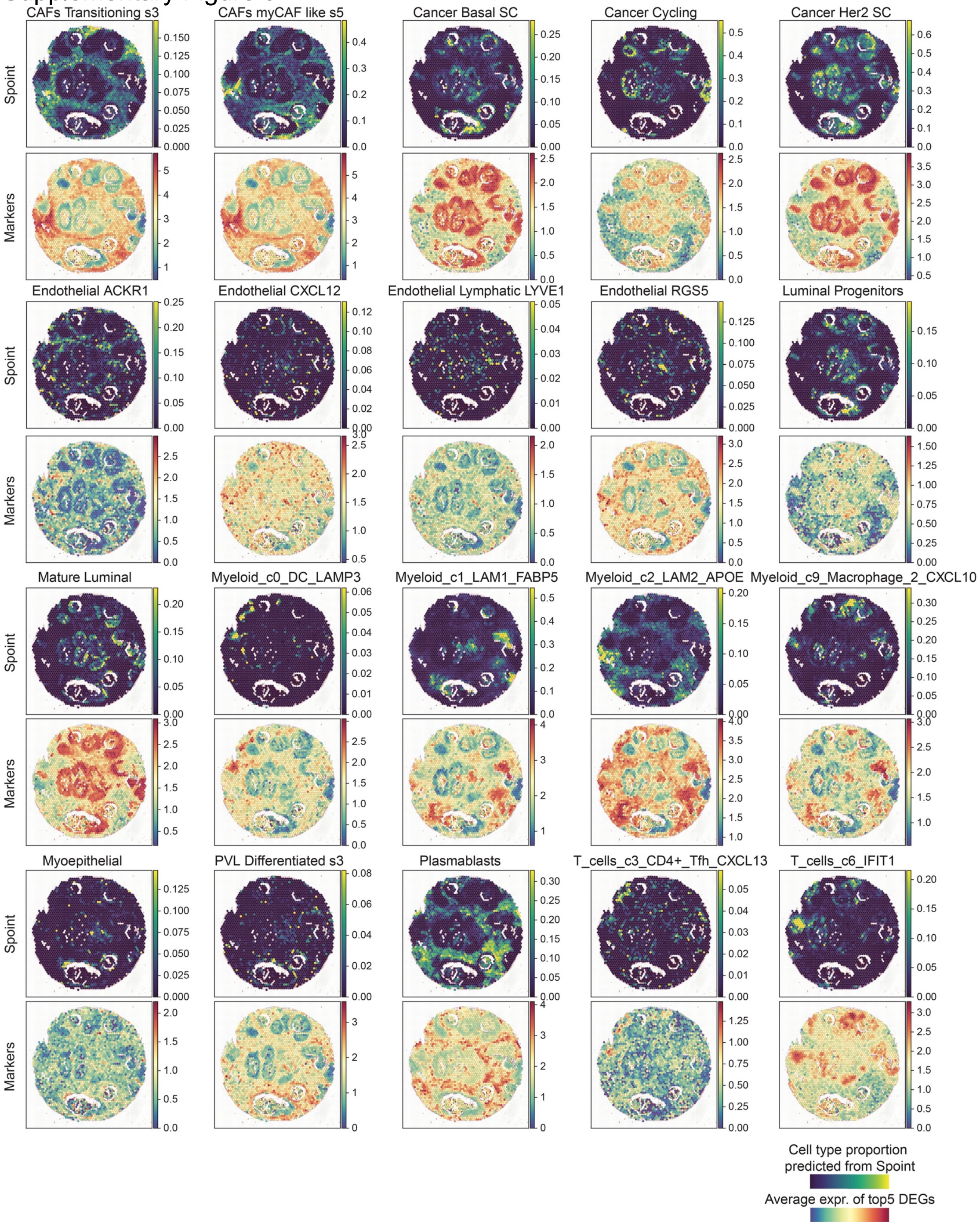

Supplementary Figure 9. Cell type composition in breast cancer dataset. Cell type compositions predicted by Spoint for breast cancer ST slice S11 from the 10X Genomics dataset (https://www.10xgenomics.com/resources/datasets). Source data are provided as a Source Data file.

Supplementary Figure 10

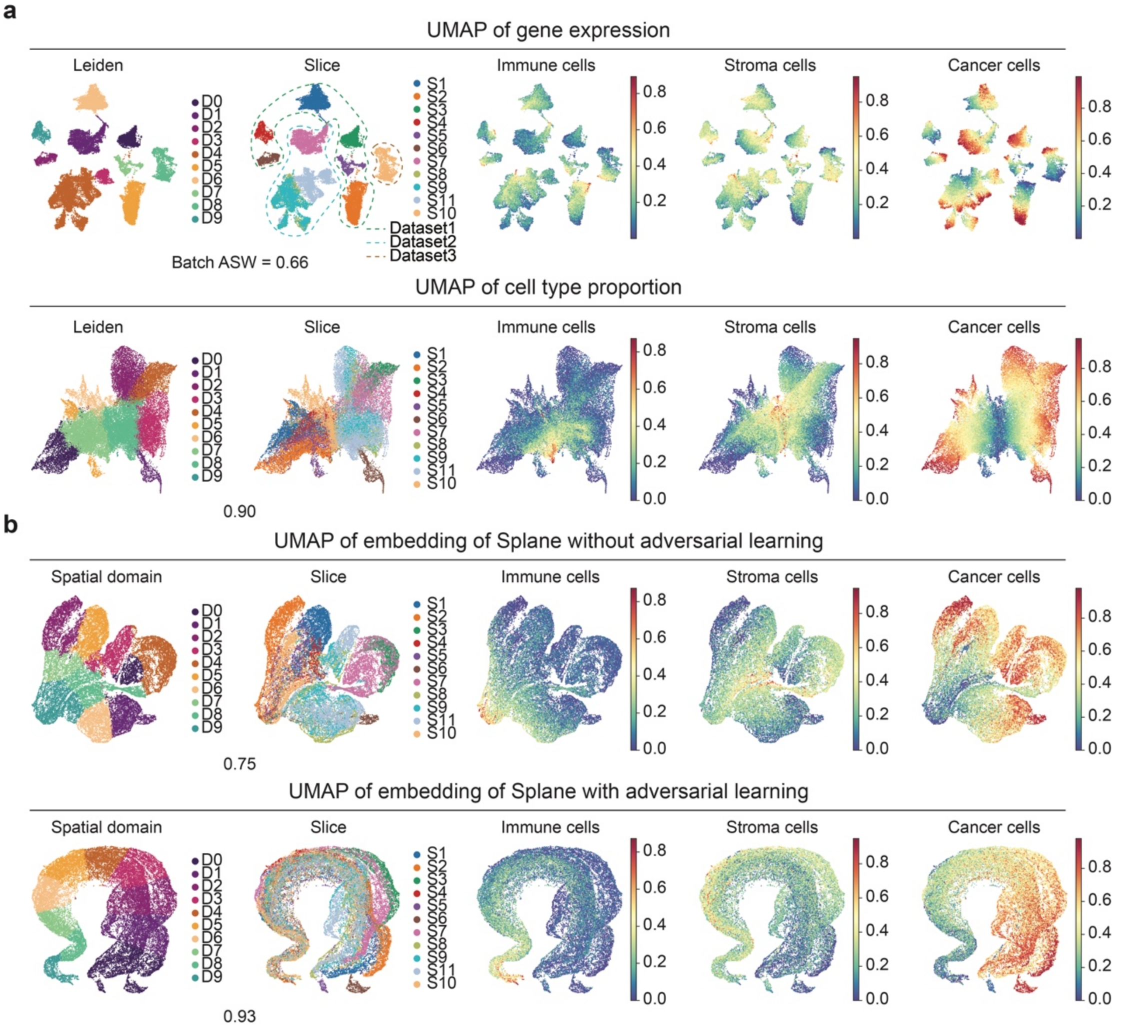

Supplementary Figure 10. Different features used for multi-slice analysis on breast cancer dataset. a, UMAP of all spots from the 11 breast cancer slices calculated from the gene expression matrix (top) and the cell type proportion predicted by Spoint (bottom) color by cluster, slice, immune cells, stroma cells, and cancer cells. UMAP, uniform manifold approximation and projection; ASW, average silhouette width. **b,** UMAP of all spots from the 11 breast cancer slices calculated from the shared latent features generated by Splane without adversarial learning (top) and the latent features generated by Splane with adversarial learning (bottom). Spots are colored by spatial domain, slice, immune cells, stroma cells, and cancer cells.

Supplementary Figure 11

a

	Tumor cells	Stroma cells	Immune cells
D0	**0.64**	0.07	0.06
D1	**0.62**	0.11	0.11
D2	**0.56**	0.17	0.13
D3	**0.47**	0.21	0.18
D4	0.39	0.20	0.26
D5	0.30	0.24	0.31
D6	0.23	0.28	0.35
D7	0.18	0.28	**0.42**
D8	0.13	0.26	**0.49**
D9	0.08	0.21	**0.60**

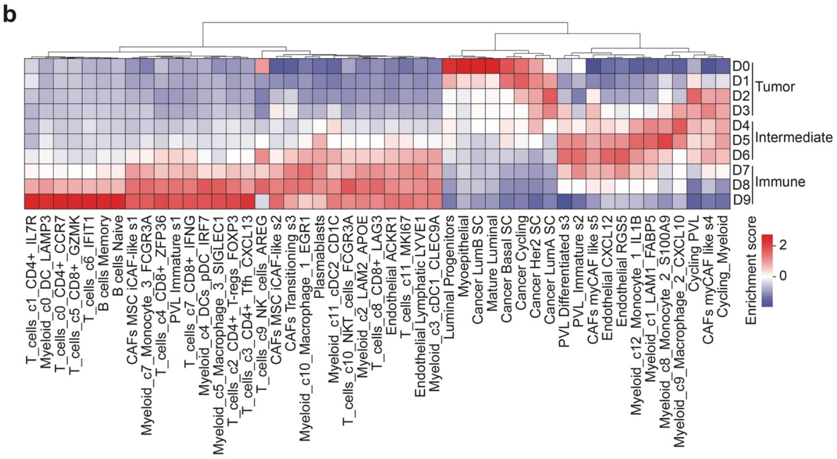

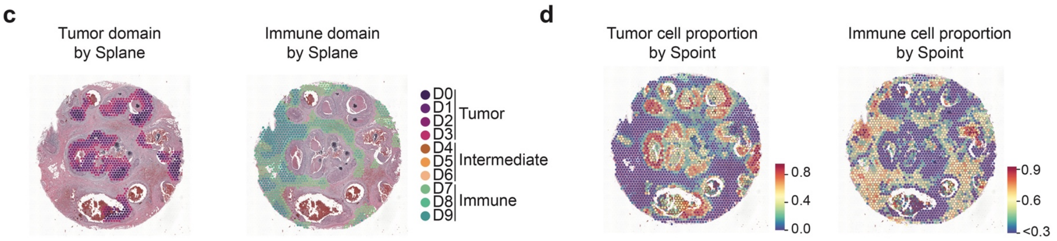

Supplementary Figure 11. Characterize spatial domains generated by Splane on breast cancer dataset. a, Proportions of tumor cells, stroma cells, and immune cells in spatial domains identified by Splane for the 11 breast cancer slices. **b,** Scaled proportion of each cell sub-type in spatial domains identified by Splane. **c,** Distributions of tumor domains and immune domains in slice S11. **d,** Proportions of tumor cells and immune cells in slice S11. Source data are provided as a Source Data file.

Supplementary Figure 12

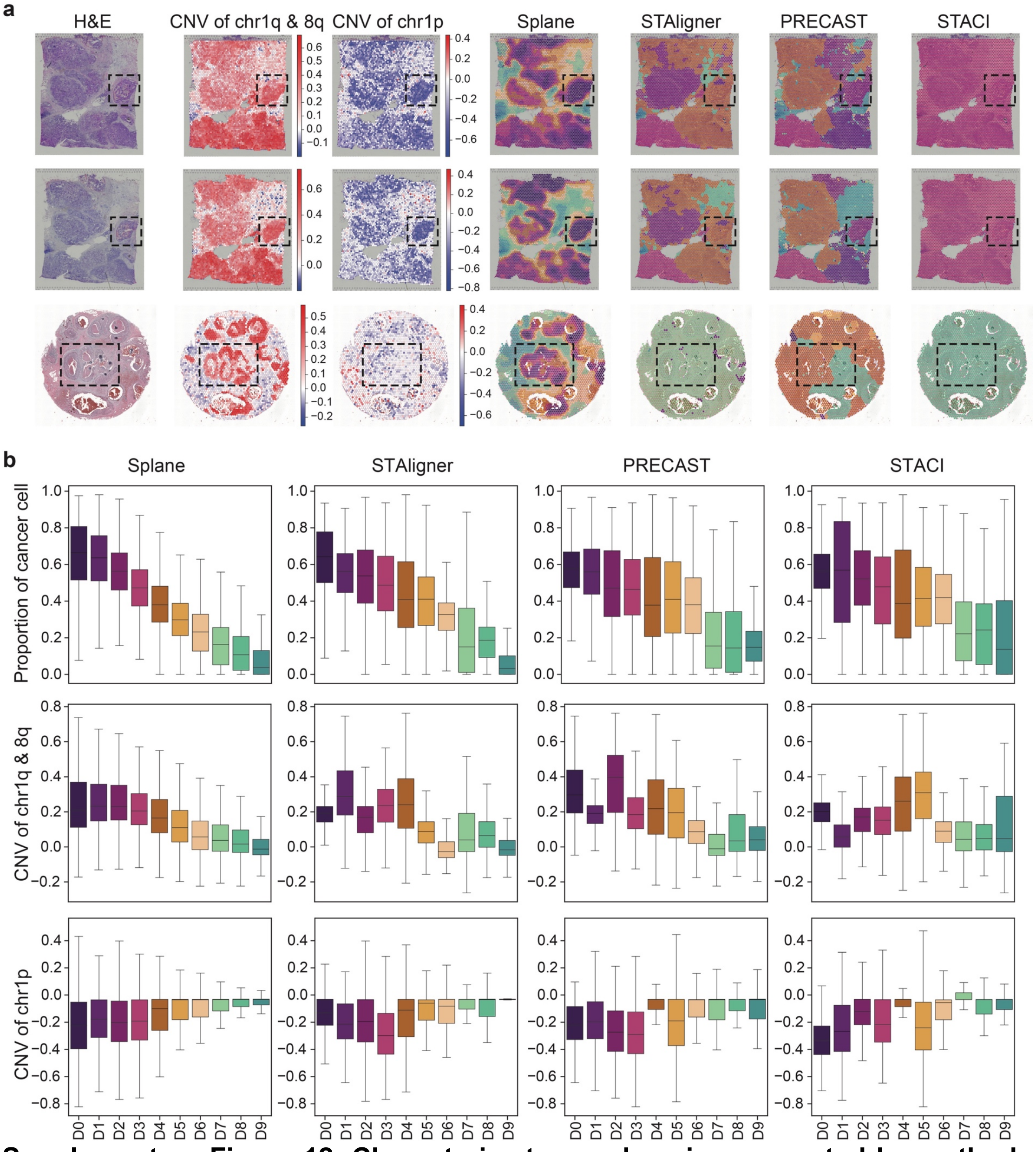

Supplementary Figure 12. Characterize tumor domains generated by methods for multi-slice analysis on breast cancer dataset. a, From left to right are the distributions of H&E staining, CNV of chromosome 1q and 8q, CNV of chromosome 1p, spatial domain generated by Splane, STAligner, PRECAST, and STACI in slice 8 (top), 9 (middle), and 11 (bottom). Dashed lines represent the positions of tumor region. CNV, copy number variations**. b,** From top to bottom are the bar plots of proportion of cancer cell, CNV of chromosome 1q and 8q, and CNV of chromosome 1p in spatial domain generated by Splane, STAligner, PRECAST, and STACI in all 11 slices. Center line, median value; box limits, upper and lower quartiles; whiskers, 1.5× interquartile range; n=11 slices. Source data are provided as a Source Data file.

Supplementary Figure 13

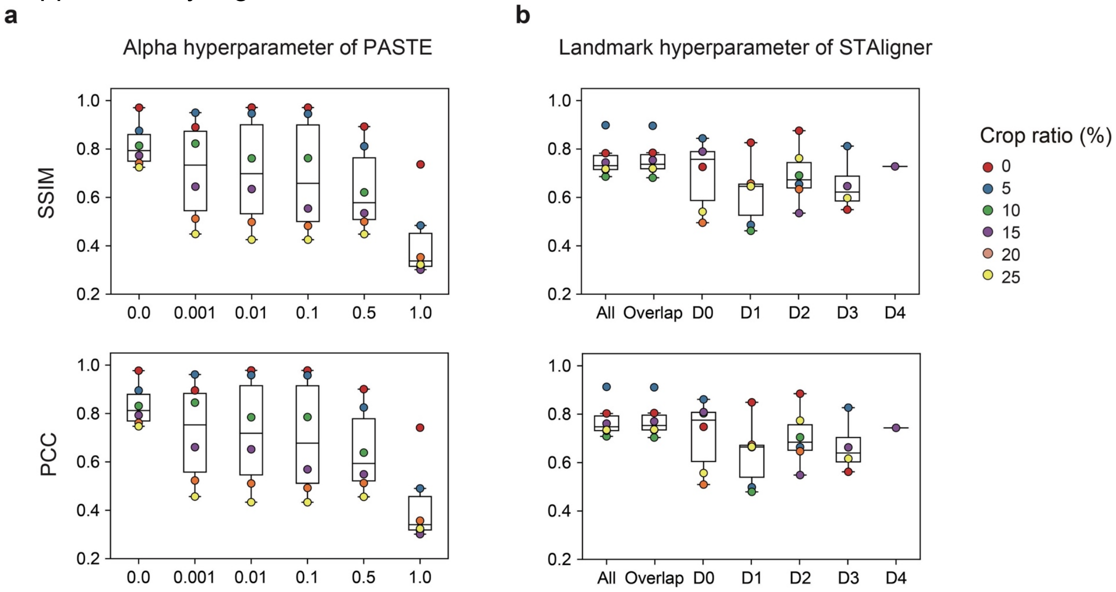

Supplementary Figure 13. Hyperparameter tuning of STAligner and PASTE for 3D alignment in STARmap dataset. a,b Optimization of the alpha hyperparameter of PASTE (a) and landmark hyperparameter of STAligner (b) for different crop ratios in STARmap dataset of the mouse brain. For each crop ratio, the hyperparameters are selected based on the best-performing results. SSIM, structural similarity index measure; PCC, pearson's correlation coefficient; center line, median value; box limits, upper and lower quartiles; whiskers, 1.5× interquartile range; n=10 slices. Source data are provided as a Source Data file.

Supplementary Figure 14

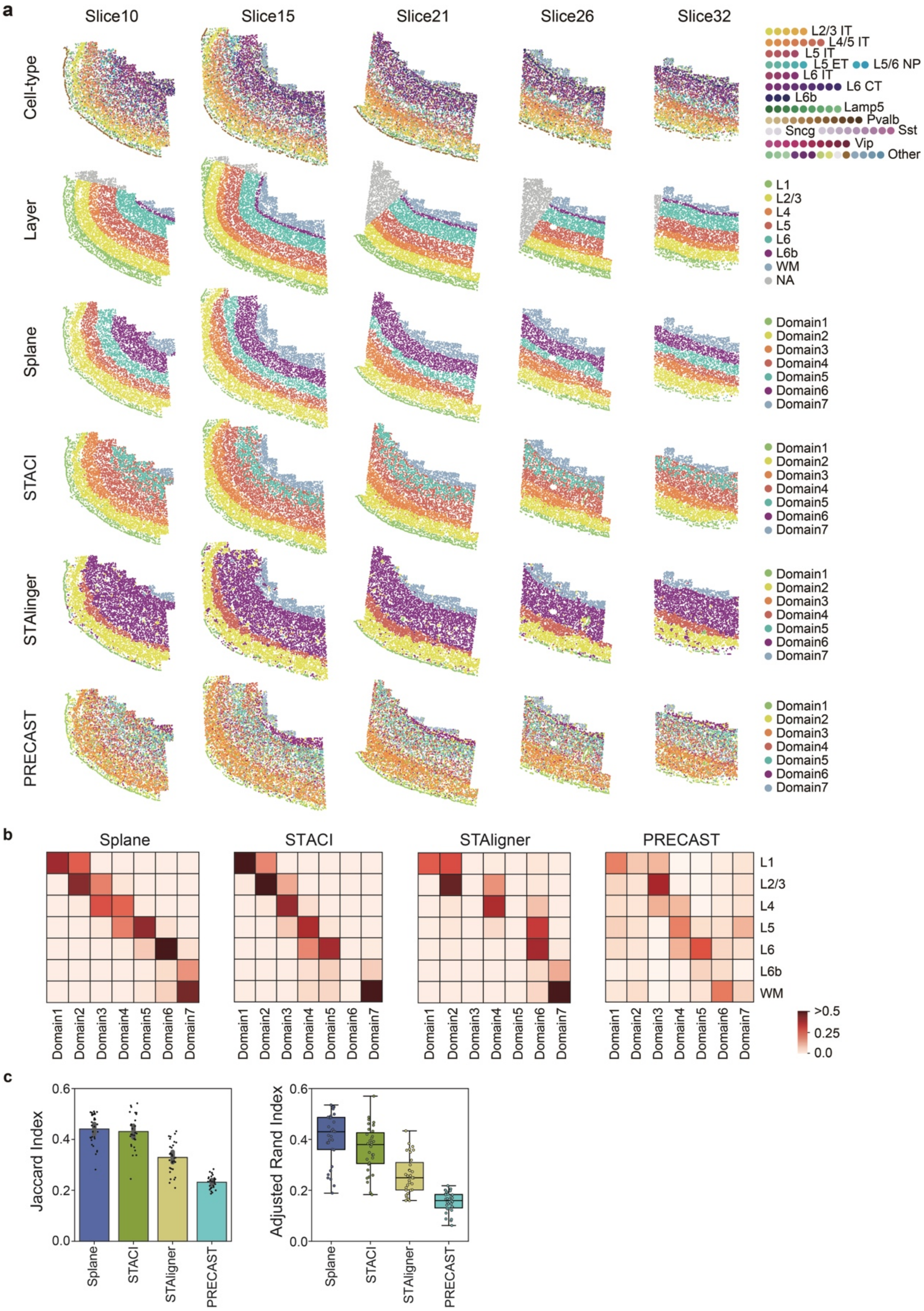

Supplementary Figure 14. Benchmarking of Splane's performance on MERFISH dataset of mouse primary motor cortex. a, Distributions of the original study-annotated cell types (Zhang *et al*. ***Nature*** 598, 137, 2021), ground truth layers, (identified following the methodology of the original study; two slices were excluded due to the lack of L6b cells, which prevented the definition of ground truth), and spatial domains identified by Splane, STAligner, PERCAST, and STACI in slice 10, 15, 21, 26, and 32. **b,** Heatmap of Jaccard indexes between the original study-annotated cortical

layers and identified spatial domains by Splane, STAligner, PERCAST, and STACI. WM, white matter. **c,** Jaccard indexes and Adjusted Rand Indexes between the original study-annotated cortical layers and identified spatial domains by Splane, STAligner, PERCAST, and STACI. Bar height, mean value; Center line, median value; box limits, upper and lower quartiles; whiskers, mean values ± 95% confidence intervals for the bar plots, 1.5× interquartile range for the box plots; n=31 slices. Source data are provided as a Source Data file.

Supplementary Figure 15

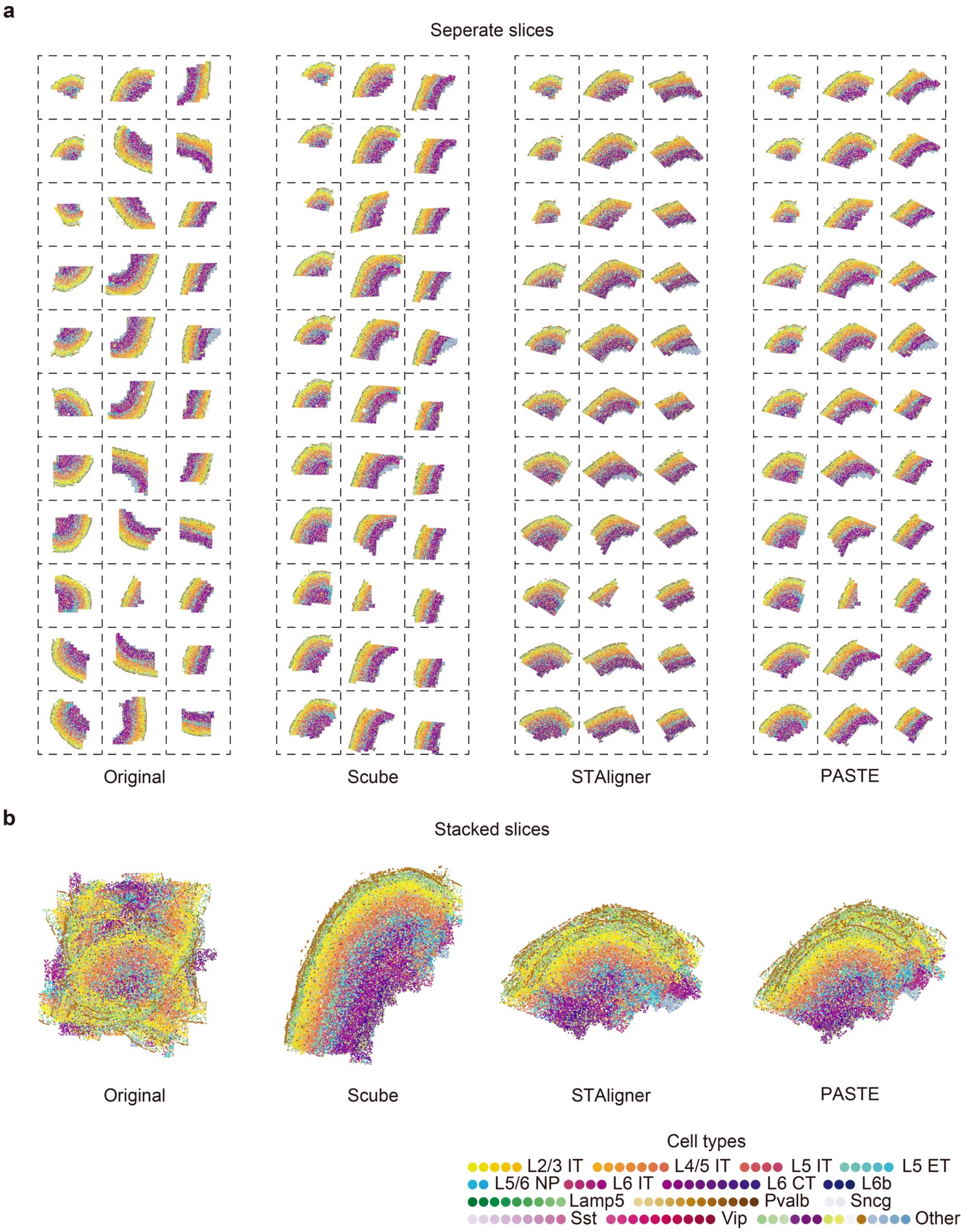

Supplementary Figure 15. Benchmarking of Scube's performance on MERFISH dataset of mouse primary motor cortex. a, From left to right are the original positions, Scube-transformed positions, STAligner-transformed positions, and PASTE-transformed positions of the 33 slices. **b,** From left to right are the slices stacked according to the original positions, the Scube-transformed positions, the STAligner-transformed positions, and the PASTE-transformed positions. Cells are labelled by cell type. Source data are provided as a Source Data file.

Supplementary Figure 16

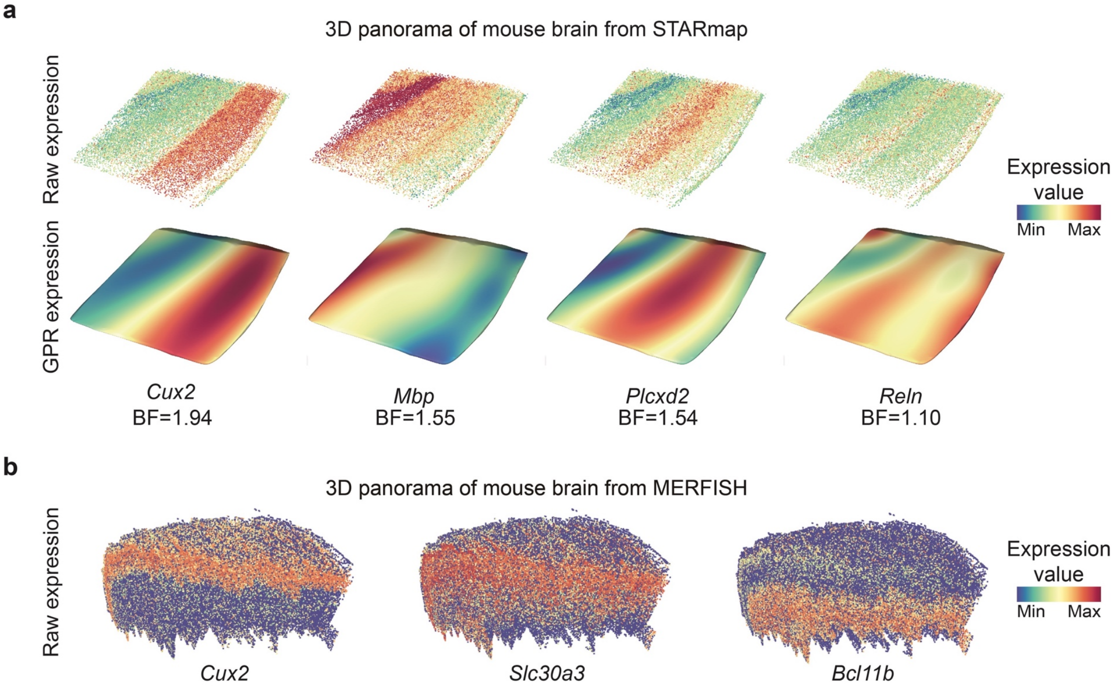

Supplementary Figure 16. Scube GPR model performance with 3D panorama. a, Raw expression distributions (upper panels) and GPR model-predicted expression distributions (lower panels) of *Cux2*, *Mbp*, *Plcxd2*, and *Reln* on the coronal plane of the 3D alignment constructed by Scube for the mouse brain STARmap dataset. BF, bayes factor. **b,** The expression distribution of *Cux2*, *Slc30a3*, and *Bcl11b* in the 3D alignment constructed by Scube for the mouse primary motor cortex MERFISH dataset.

Supplementary Figure 17

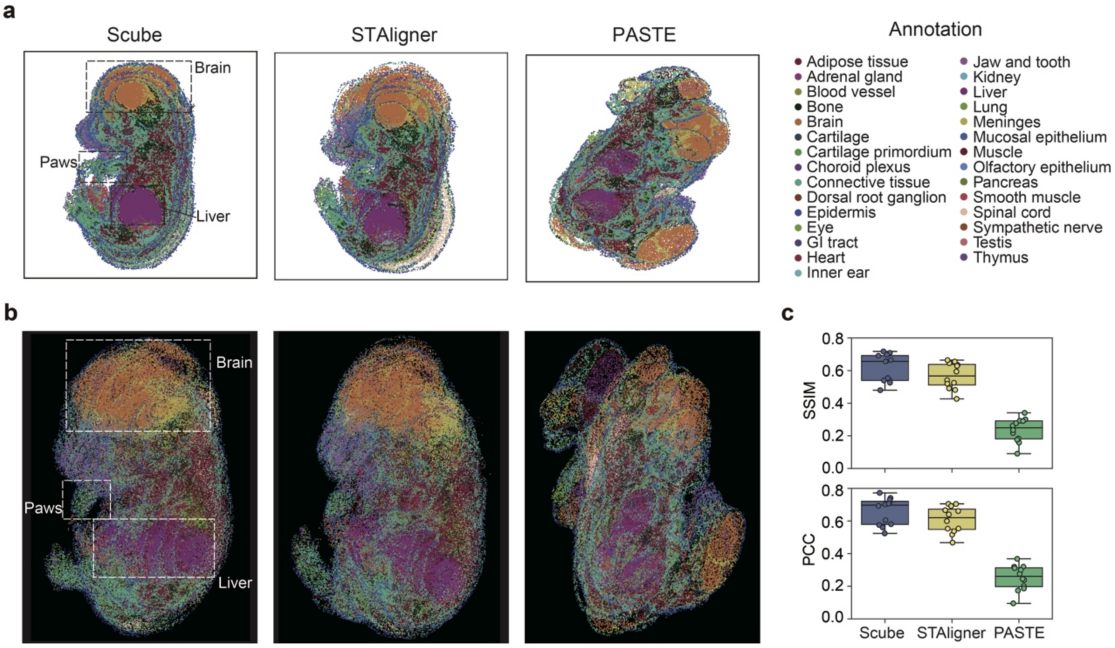

Supplementary Figure 17. Analysis mouse embryo Stereo-seq data with integrated workflow of SPACEL. a,b, The slices constructed by Scube, STAligner, and PASTE stacked in (a) 2D and (b) 3D. The dashed boxes indicate the reconstructed structures of the brain, paw, and liver. Cells are labeled based on their cell type annotations from the original study (Chen et al. ***Cell***, 2022, 185, 1777). **c,** SSIM/PCC values of Scube's, STAligner's, and PASTE's alignment results for the Stereo-seq dataset. SSIM, structural similarity index measure; PCC, pearson's correlation coefficient; center line, median value; box limits, upper and lower quartiles; whiskers, 1.5× interquartile range; n=13 slices. Source data are provided as a Source Data file.

Supplementary Figure 18

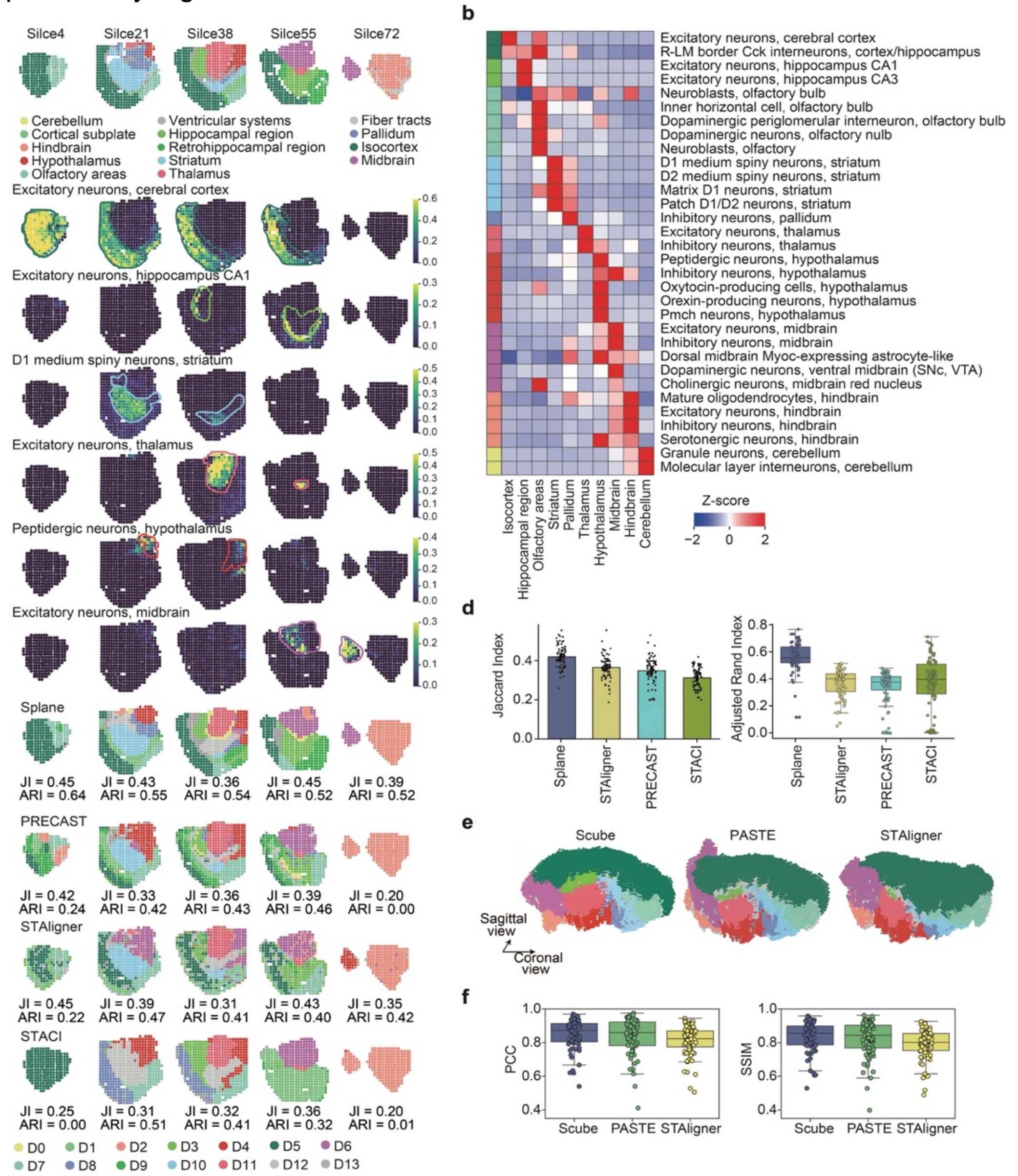

Supplementary Figure 18. Analysis of mouse whole brain data with integrated workflow of SPACEL. a, Spatial annotations of brain regions in slice 4, 21, 38, 55, and 72 from the mouse brain dataset generated by Spatial Transcriptomics technology (Cantin Ortiz et al. ***Sci. Adv.*** 6, 2020), and cell type compositions predicted by Spoint. **b,** Scaled proportion of each cell type predicted by Spoint in each region. Cell types are filtered based on having a maximum proportion greater than 0.4 across all spots. **c,** Spatial domains identified by SPACEL, STAligner, PERCAST, and STACI. JI, Jaccard index; ARI, Adjusted Rand Index. **d,** Jaccard indexes and Adjusted Rand Indexes between brain region annotations and corresponding spatial domains identified by Splane, STAligner, PERCAST, and STACI. Bar height, mean value; center line, median value; box limits, upper and lower quartiles; whiskers, mean values ± 95% confidence intervals for the bar plots, 1.5× interquartile range for the box plots; n=75 slices. **e,** Stacked 3D alignments constructed by Scube, PASTE, and STAligner from 75 slices at 40 different z-coordinates of mouse brain. **f,** SSIM/PCC values of Scube's, PASTE's, and STAligner's alignment results for the dataset. SSIM, structural similarity index measure; PCC, pearson's correlation coefficient; center line, mean value; box limits, upper and lower quartiles; whiskers, 1.5× interquartile range; n=75 slices. Source data are provided as a Source Data file.
