# Supplementary material for: SPACEL: deep learning-based characterization of spatial transcriptome architectures
Source: Nat Commun. 2023 Nov 22;14:7603. doi: 10.1038/s41467-023-43220-3 (PMC10663563; doi:10.1038/s41467-023-43220-3)
Supplement: Supplementary file 3 — Description of Additional Supplementary Files [file 41467_2023_43220_MOESM3_ESM.pdf]

Description of Additional Supplementary Files:

Supplementary Data 1: Detailed information of six spatial transcriptomics datasets and two paired scRNA-seq datasets.

Supplementary Data 2: The selection of hyperparameters for each method.

Supplementary Data 3: Categories of cell types in breast cancer scRNA-seq dataset.
