# Supplementary material for: SPACEL: deep learning-based characterization of spatial transcriptome architectures
Source: Nat Commun. 2023 Nov 22;14:7603. doi: 10.1038/s41467-023-43220-3 (PMC10663563; doi:10.1038/s41467-023-43220-3)
Supplement: Supplementary file 7 — Reporting Summary [file 41467_2023_43220_MOESM7_ESM.pdf]

nature portfolio

NCOMMS-22-53369B
Corresponding author(s): Kun QU
qukun@ustc.edu.cn

Last updated by author(s): Oct 10, 2023

Reporting Summary

Nature Portfolio wishes to improve the reproducibility of the work that we publish. This form provides structure for consistency and transparency in reporting. For further information on Nature Portfolio policies, see our Editorial Policies and the Editorial Policy Checklist.

Statistics

For all statistical analyses, confirm that the following items are present in the figure legend, table legend, main text, or Methods section.

n/a	Confirmed	
☐	☒	The exact sample size (n) for each experimental group/condition, given as a discrete number and unit of measurement
☐	☒	A statement on whether measurements were taken from distinct samples or whether the same sample was measured repeatedly
☐	☒	The statistical test(s) used AND whether they are one- or two-sided<br>*Only common tests should be described solely by name; describe more complex techniques in the Methods section.*
☐	☒	A description of all covariates tested
☐	☒	A description of any assumptions or corrections, such as tests of normality and adjustment for multiple comparisons
☐	☒	A full description of the statistical parameters including central tendency (e.g. means) or other basic estimates (e.g. regression coefficient) AND variation (e.g. standard deviation) or associated estimates of uncertainty (e.g. confidence intervals)
☐	☒	For null hypothesis testing, the test statistic (e.g. F, t, r) with confidence intervals, effect sizes, degrees of freedom and P value noted<br>*Give P values as exact values whenever suitable.*
☒	☐	For Bayesian analysis, information on the choice of priors and Markov chain Monte Carlo settings
☒	☐	For hierarchical and complex designs, identification of the appropriate level for tests and full reporting of outcomes
☐	☒	Estimates of effect sizes (e.g. Cohen's d, Pearson's r), indicating how they were calculated

Our web collection on statistics for biologists contains articles on many of the points above.

Software and code

Policy information about availability of computer code

Data collection	No software was used for data collection.
Data analysis	We compared the performance of the Spoint module of SPACEL with 11 deconvolution methods: CARD(Version 1.0), Cell2location(Version 0.6a0), DestVI(0.14.4), SPOTlight(1.0.1), SpatialDWLS(Version 1.1.2), Seurat(Version 4.0.5), Tangram(1.0.0), RCTD(Version 1.2.0), Stereoscope (Version 0.14.4), STRIDE(Version 0.0.1b0), and DSTG (Version 0.0.1).<br>We compared the performance of the Splane module of SPACEL with 7 spatial-domain-identification methods: STAGATE (Version 1.0.1), SpaGCN (Version 1.2.5), BayersSpace (Version 1.0.0), stLearn (Version 0.4.7), STAligner (Version 1.0.0), PRECAST (Version 1.6), and STACI (Version 0).<br>We compared the performance of the Scube module of SPACEL with PASTE (Version 1.3.0) and STAligner (Version 1.0.0).<br>The SPACEL open source package is available at a GitHub repository: https://github.com/QuKunLab/SPACEL. We uploaded the code and scripts used for the analysis and figure plotting to a public Zenodo repository (https://doi.org/10.5281/zenodo.8316334).

For manuscripts utilizing custom algorithms or software that are central to the research but not yet described in published literature, software must be made available to editors and reviewers. We strongly encourage code deposition in a community repository (e.g. GitHub). See the Nature Portfolio guidelines for submitting code & software for further information.

Data

Policy information about availability of data

All manuscripts must include a data availability statement. This statement should provide the following information, where applicable:

- Accession codes, unique identifiers, or web links for publicly available datasets
- A description of any restrictions on data availability
- For clinical datasets or third party data, please ensure that the statement adheres to our policy

The simulated datasets used for the evaluation of Spoint and other deconvolution methods are available at: https://github.com/QuKunLab/SPACEL.
All spatial transcriptomics and single-cell RNA-seq datasets used in this study can be downloaded from public websites and/or databases:
(1) 12 slices of human DLPFC Visium data are available at http://research.libd.org/spatialLIBD/;
(2) 6 slices of human breast cancer Visium data are available at https://doi.org/10.5281/zenodo.4739739;
(3) 4 slices of human breast cancer Visium data are available at https://support.10xgenomics.com/spatial-gene-expression/datasets, including samples "Parent_Visium_Human_BreastCancer", "V1_Breast_Cancer_Block_A_Section_1", "V1_Breast_Cancer_Block_A_Section_2", and "Visium_FFPE_Human_Breast_Cancer";
(4) one slice of human breast cancer Visium data is available at https://support.10xgenomics.com/spatial-gene-expression/datasets, termed as "Invasive Ductal Carcinoma Stained With Fluorescent CD3 Antibody";
(5) Mouse brain STARmap data are available at https://www.starmapresources.org/data;
(6) 33 slices of Mouse MOp MERFISH data are available at https://doi.brainimagelibrary.org/doi/10.35077/g.21;
(7) one slice of mouse E16.5 embryo brain Stereo-seq data, one slice of mouse brain Stereo-seq data, and 13 slices of mouse E16.5 whole embryo Stereo-seq data are available at https://db.cngb.org/stomics/mosta/download/;
(8) ten slice of human brain MERFISH data are available at https://datadryad.org/stash/dataset/doi:10.5061/dryad.x3ffbg7mw;
(9) 75 slice of mouse whole brain Spatial Transcriptomics data are available using GEO accession number GSE147747;
(10) single-nucleus transcriptomies data across multiple human cortical areas are available at https://portal.brain-map.org/atlases-and-data/rnaseq/human-multiple-cortical-areas-smart-seq;
(11) single-cell transcriptomies data of human breast cancer data are available at https://singlecell.broadinstitute.org/single_cell/study/SCP1039;
(12) single-cell transcriptomics data of mouse embryo brain are available at http://mousebrain.org/development/downloads.html;
(13) single-cell transcriptomics data of mouse whole cortex and hippocampus are available at https://portal.brain-map.org/atlases-and-data/rnaseq/mouse-whole-cortex-and-hippocampus-10x;
(14) single-cell transcriptomics data of mouse whole brain are available at mousebrain.org/adolescent/downloads.html.
We also provide a public Zenodo repository for users to download all the above datasets (https://doi.org/10.5281/zenodo.8316334).

Research involving human participants, their data, or biological material

Policy information about studies with human participants or human data. See also policy information about sex, gender (identity/presentation), and sexual orientation and race, ethnicity and racism.

Reporting on sex and gender	N/A
Reporting on race, ethnicity, or other socially relevant groupings	N/A
Population characteristics	N/A
Recruitment	N/A
Ethics oversight	N/A

Note that full information on the approval of the study protocol must also be provided in the manuscript.

Field-specific reporting

Please select the one below that is the best fit for your research. If you are not sure, read the appropriate sections before making your selection.

☒ Life sciences ☐ Behavioural & social sciences ☐ Ecological, evolutionary & environmental sciences

For a reference copy of the document with all sections, see nature.com/documents/nr-reporting-summary-flat.pdf

Life sciences study design

All studies must disclose on these points even when the disclosure is negative.

Sample size	We used 11 spatial transcriptomics and 5 scRNA-seq datasets from published studies. The details of these datasets are listed as follows:<br>(1) 12 slices from human DLPFC Visium data: slice 1, 4,226 spots; slice 2, 4,384 spots ; slice 3, 4,789 spots; slice 4, 4,634 spots; slice 5, 3,661 spots; slice 6, 3,498 spots; slice 7, 4,110 spots; slice 8, 4,015 spots; slice 9, 3,639 spots; slice 10, 3,673 spots; slice 11, 3,592 spots; slice 12, 3,460 spots;

(2) 6 slices from human breast cancer Visium dataset: slice 1, 4,784 spots; slice 2, 2,432 spots; slice 3, 2,432 spots; slice 4, 1,211 spots; slice 5, 1,163; slice 6, 1,127 spots;
(3) 4 slices from human breast cancer Visium dataset: slice 1, 4,325 spots; slice 2, 3,798 spots; slice 3, 3,987 spots; slice 4, 4,727 spots;
(4) one slices from human breast cancer Visium dataset: 2518 spots;
(5) Mouse brain STARmap data: 32,841 cells;
(6) 33 slices from Mouse MOp MERFISH dataset: slice 1, 2378 cells; slice 2, 2423 cells; slice 3, 2033 cells; slice 4, 3106 cells; slice 5, 4159 cells; slice 6, 4359 cells; slice 7, 4792 cells; slice 8, 5749 cells; slice 9, 5262 cells; slice 10, 5661 cells; slice 11, 6731 cells; slice 12, 4996 cells; slice 13, 6185 cells; slice 14, 5061 cells; slice 15, 7626 cells; slice 16, 6963 cells; slice 17, 6459 cells; slice 18, 6264 cells; slice 19, 5730 cells; slice 20, 2197 cells; slice 21, 6218 cells; slice 22, 6535 cells; slice 23, 5763 cells; slice 24, 5375 cells; slice 25, 4052 cells; slice 26, 4429 cells; slice 27, 5745 cells; slice 28, 3899 cells; slice 29, 4308 cells; slice 30, 4453 cells; slice 31, 4408 cells; slice 32, 3920 cells; slice 33, 4145 cells;
(7) one slice from mouse E16.5 embryo brain Stereo-seq dataset: 65,303 spots;
(8) one slice from mouse brain Stereo-seq dataset: 50,140 spots;
(9) 13 slices from mouse E16.5 whole embryo Stereo-seq dataset: slice 1, 7,390 spots; slice 2, 9,238 spots; slice 3, 13,826 spots; slice 4, 12,401 spots; slice 5, 16,558 spots; slice 6, 17,148 spots; slice 7, 19,389 spots; slice 8, 15,105 spots; slice 9, 16,178 spots; slice 10, 14,249 spots; slice 11, 13,674 spots; slice 12, 11,839 spots; slice 13, 9,716 spots;
(10) ten slices from human brain MERFISH dataset: slice 1, 3,044 spots; slice 2, 4,045 spots; slice 3, 3,970 spots; slice 4, 4,835 spots; slice 5, 5,610 spots; slice 6, 6,135 spots; slice 7, 4,871 spots; slice 8, 3,918 spots; slice 9, 4,321 spots; slice 10, 3,512 spots;
(11) 75 slices from mouse whole brain Spatial Transcriptomics dataset: slice 1, 152 spots; slice 2, 197 spots; slice 3, 240 spots; slice 4, 218 spots; slice 5, 269 spots; slice 6, 274 spots; slice 7, 287 spots; slice 8, 326 spots; slice 9, 361 spots; slice 10, 357 spots; slice 11, 393 spots; slice 12, 403 spots; slice 13, 488 spots; slice 14, 461 spots; slice 15, 381 spots; slice 16, 470 spots; slice 17, 491 spots; slice 18, 512 spots; slice 19, 487 spots; slice 20, 522 spots; slice 21, 494 spots; slice 22, 506 spots; slice 23, 546 spots; slice 24, 433 spots; slice 25, 509 spots; slice 26, 580 spots; slice 27, 589 spots; slice 28, 556 spots; slice 29, 546 spots; slice 30, 560 spots; slice 31, 519 spots; slice 32, 580 spots; slice 33, 603 spots; slice 34, 577 spots; slice 35, 570 spots; slice 36, 591 spots; slice 37, 604 spots; slice 38, 556 spots; slice 39, 536 spots; slice 40, 620 spots; slice 41, 576 spots; slice 42, 589 spots; slice 43, 604 spots; slice 44, 574 spots; slice 45, 639 spots; slice 46, 617 spots; slice 47, 606 spots; slice 48, 508 spots; slice 49, 548 spots; slice 50, 534 spots; slice 51, 508 spots; slice 52, 494 spots; slice 53, 460 spots; slice 54, 468 spots; slice 55, 462 spots; slice 56, 450 spots; slice 57, 527 spots; slice 58, 548 spots; slice 59, 524 spots; slice 60, 484 spots; slice 61, 490 spots; slice 62, 478 spots; slice 63, 479 spots; slice 64, 428 spots; slice 65, 419 spots; slice 66, 386 spots; slice 67, 406 spots; slice 68, 237 spots; slice 69, 250 spots; slice 70, 279 spots; slice 71, 276 spots; slice 72, 245 spots; slice 73, 240 spots; slice 74, 188 spots; slice 75, 214 spots;
(12) single-nucleus transcriptomes data across multiple human cortical areas: 47,432 cells;
(13) single-cell transcriptomies data of human breast cancer data: 100,064 cells;
(14) single-cell transcriptomics data of mouse embryo brain: 292,495 cells;
(15) single-cell transcriptomics data of mouse whole cortex and hippocampus: 1,169,213 cells;
(16) single-cell transcriptomics data of mouse whole brain: 135,637 cells.
No statistical method was used to predetermine sample size. These samples are adequate as they contain multiple tissues and states from both human and mouse.

Data exclusions

In Fig. 2a&b, the CARD was excluded from the benchmark on the scRNA-seq data simulated datasets due to lack spatial information for these datasets. In Supplementary Fig. 4e, the STRIDE was not shown in Stereo-seq mouse embryo brain dataset due to a runtime error for this dataset. In Supplementary Fig. 14a, two slices were excluded due to the lack of L6b cells, which prevented the definition of ground truth.

Replication

To make sure that the experimental findings are reproducible, we (1) compared the performance of the Spoint module of SPACEL with 11 deconvolution methods for predicting the spatial distribution of undetected transcripts on 32 simulated datasets, the 12 slices from human DLPFC 10X Visium dataset and three real spatial transcriptomic dataset with single-cell resolution, (2) compared the performance of the Splane module of SPACEL with 7 spatial-domain-identification methods using the 12 slices from human DLPFC 10X Visium dataset, the 11 slices from three different human breast cancer 10X Visium datasets, the 33 slices from mouse MOp MERFISH dataset and the 75 slices from mouse whole brain Spatial Transcriptomics dataset. (3) compare the performance of the Scube module of SPACEL with PASTE and STAligner in the 3D alignment construction of the mouse brain STARmap dataset, the 33 slices from mouse MOp MERFISH dataset, the 13 slices from mouse E16.5 whole embryo Stereo-seq dataset and the 75 slices from mouse whole brain Spatial Transcriptomics dataset. All these replications successfully reproduced our findings.

Randomization

The experiments were not randomized, because we used all collected data for analysis.

Blinding

The Investigators were not blinded to allocation during experiments and outcome assessment.

Reporting for specific materials, systems and methods

We require information from authors about some types of materials, experimental systems and methods used in many studies. Here, indicate whether each material, system or method listed is relevant to your study. If you are not sure if a list item applies to your research, read the appropriate section before selecting a response.

Materials & experimental systems

n/a	Involved in the study
☒	☐ Antibodies
☒	☐ Eukaryotic cell lines
☒	☐ Palaeontology and archaeology
☒	☐ Animals and other organisms
☒	☐ Clinical data
☒	☐ Dual use research of concern
☒	☐ Plants

Methods

n/a	Involved in the study
☒	☐ ChIP-seq
☒	☐ Flow cytometry
☒	☐ MRI-based neuroimaging

Plants

Seed stocks

Report on the source of all seed stocks or other plant material used. If applicable, state the seed stock centre and catalogue number. If plant specimens were collected from the field, describe the collection location, date and sampling procedures.

Novel plant genotypes

Describe the methods by which all novel plant genotypes were produced. This includes those generated by transgenic approaches, gene editing, chemical/radiation-based mutagenesis and hybridization. For transgenic lines, describe the transformation method, the number of independent lines analyzed and the generation upon which experiments were performed. For gene-edited lines, describe the editor used, the endogenous sequence targeted for editing, the targeting guide RNA sequence (if applicable) and how the editor was applied.

Authentication

Describe any authentication procedures for each seed stock used or novel genotype generated. Describe any experiments used to assess the effect of a mutation and, where applicable, how potential secondary effects (e.g. second site T-DNA insertions, mosiacism, off-target gene editing) were examined.
